# An enhanced whale optimization algorithm for task scheduling in edge computing environments

**DOI:** 10.3389/fdata.2024.1422546

**Published:** 2024-10-30

**Authors:** Li Han, Shuaijie Zhu, Haoyang Zhao, Yanqiang He

**Affiliations:** College of Computer Science and Technology, Zhengzhou University of Light Industry, Zhengzhou, China

**Keywords:** multi-objective optimization, whale optimization algorithm, task scheduling, edge computing, optimization in edge computing

## Abstract

The widespread use of mobile devices and compute-intensive applications has increased the connection of smart devices to networks, generating significant data. Real-time execution faces challenges due to limited resources and demanding applications in edge computing environments. To address these challenges, an enhanced whale optimization algorithm (EWOA) was proposed for task scheduling. A multi-objective model based on CPU, memory, time, and resource utilization was developed. The model was transformed into a whale optimization problem, incorporating chaotic mapping to initialize populations and prevent premature convergence. A nonlinear convergence factor was introduced to balance local and global search. The algorithm's performance was evaluated in an experimental edge computing environment and compared with ODTS, WOA, HWACO, and CATSA algorithms. Experimental results demonstrated that EWOA reduced costs by 29.22%, decreased completion time by 17.04%, and improved node resource utilization by 9.5%. While EWOA offers significant advantages, limitations include the lack of consideration for potential network delays and user mobility. Future research will focus on fault-tolerant scheduling techniques to address dynamic user needs and improve service robustness and quality.

## 1 Introduction

**Figure 1:**
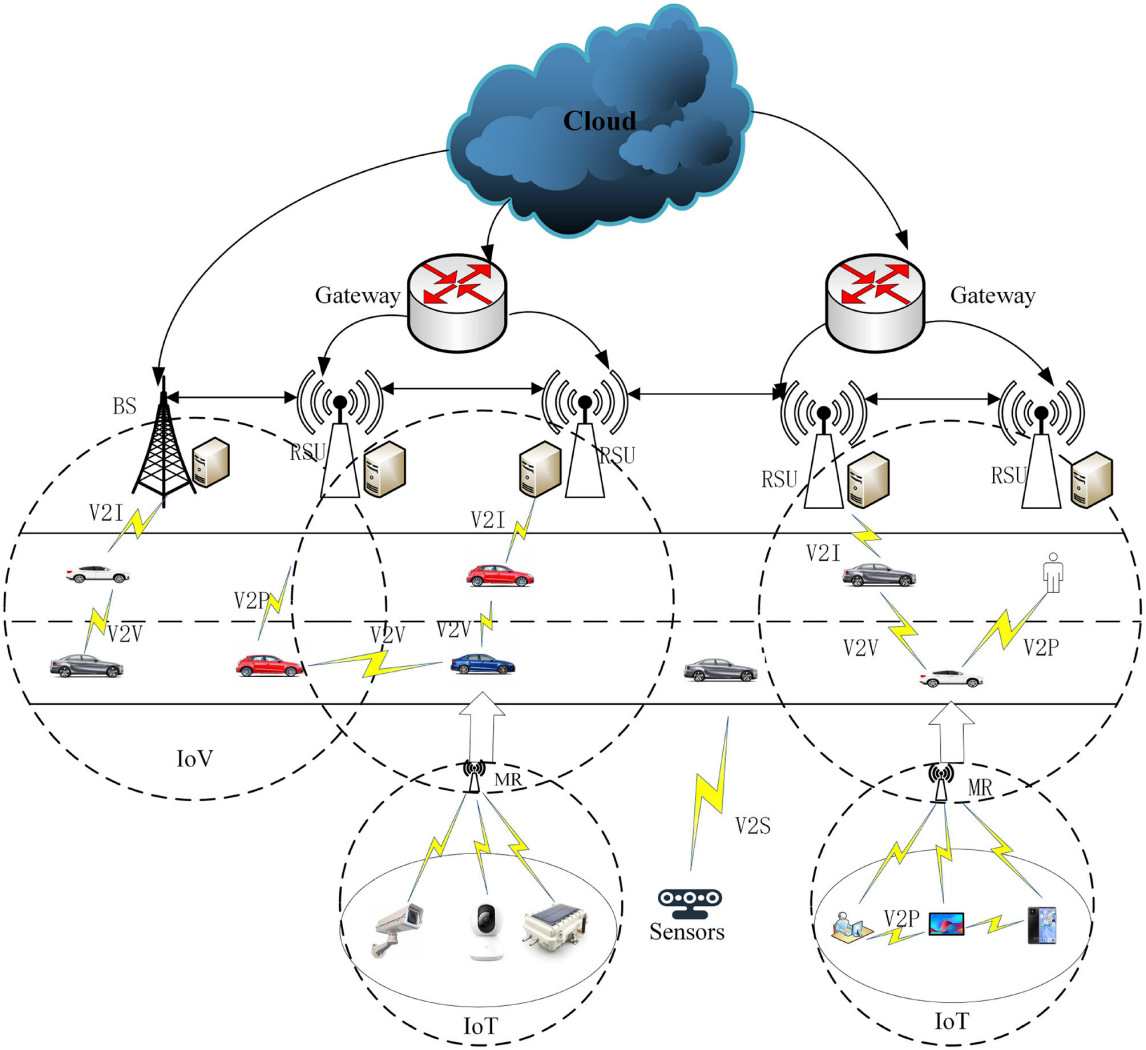
Application scenarios for computationally intensive task scheduling in edge environments.

Based on the Ericsson Mobile Report (Cisco, 2023), in the third quarter of 2023, 163 million new 5G subscriptions were added, reaching a total of 1.4 billion. Mobile network data traffic experienced a 33% growth between Q3 2022 and Q3 2023. By the end of 2029, it is anticipated that 5G mobile subscriptions to surpass 5.3 billion, and the average global mobile data consumption per smartphone is expected to reach 56 GB per month. In order to develop 5G wireless technology and enable network transmission with exceptionally high rates of data, extremely low latency, minimal energy usage, an outstanding experience, and the security of user data (Ghobaei-Arani et al., 2020), edge computing is proposed as an efficient solution (Li et al., 2021). In edge computing, overloaded computation tasks can be scheduled by edge devices to the edge servers. This enables the full utilization of edge servers in terms of computation and storage, ultimately enhancing the delivery of real-time services. In edge computing, the critical problem is selecting the appropriate edge server for the computation requester based on the network state and edge server. The crucial factor in selecting the right edge server depends on whether the applicant, equipped with the compute function, comprehends the services available in its vicinity (Zhao et al., 2021). Edge computing apps depend on an administrative server for proxies to choose the right side server. Task scheduling plays an important part in increasing resource utilization and providing high-quality services to users. Task scheduling techniques have surfaced as one of the most popular topics in edge computing (Hazra et al., 2020). An effective task scheduling strategy can adapt flexibly to the dynamic edge and cloud computing environments, successfully decreasing the time needed for users to submit assignments and enhancing the efficiency of resource utilization (Barika et al., 2021). In edge computing environments, task scheduling has found extensive applications in scenarios such as the Internet of Things (IoT), the Internet of Vehicles (IoV), and intelligent transportation. The utilization of edge computing scheduling not only addresses the constraints of the existing host network but also plays a crucial role in achieving efficient task allocation Figure 1 illustrates the application scenarios of compute-intensive task scheduling in edge environments. In Figure 1, the Internet of Vehicles (IoV) supports various communication modes, including vehicle-to-sensor (V2S), vehicle-to-infrastructure (V2I), and vehicle-to-vehicle (V2V). In this setup, vehicles connect to Roadside Units (RSUs) of matched edge servers to execute real-time or critical computational tasks. Service users, such as vehicles and terminals, can access computational and storage services from the Edge Computing Servers (ECSs) of base stations (BSs) located in their proximity. When a vehicle user submits a task to be scheduled by the base station, the scheduling algorithm determines whether to schedule the computation task to the service queue at the node end of the base station or to the vehicle service queue. Subsequently, the Scheduling Strategy for the task is implemented by the edge server, and then it returns to the user.

In recent years, task scheduling has become a significant research topic. The main challenge faced by edge cloud task scheduling technology is determining the optimal strategy for allocating resources to nodes. This is crucial for meeting consumers' QoS expectations and enhancing overall server performance. Numerous studies have introduced task scheduling strategies; however, the majority predominantly focus on factors like execution time or task scheduling cost. Notably, there is a scarcity of attention given to issues such as server-side resource overloading, which arises from the concurrent processing of large volumes of computationally intensive data. The innovation of this paper lies in proposing a task scheduling strategy that integrates user QoS requirements with server resource node characteristics. The aim is to improve resource utilization, reduce scheduling time and cost, optimize the configuration of container resource nodes, enhance user experience, and effectively address the issue of resource overload. Specifically, this paper focuses on task resource utilization, cost, and execution time within an edge computing system for a given task. These steps are crucial to ensuring that the configuration of container resource nodes is optimized as much as possible.

The problem is defined as follows: in an edge computing environment, given a task set *I* = {*I*_1_, *I*_2_, *I*_3_⋯*I*_*i*_}, and a resource node set *R* = {*R*_1_, *R*_2_, *R*_3_, ⋯ , *R*_*r*_}, where each task *i* has specific resource requirements (e.g., CPU, memory, bandwidth), and each resource node *r* has limited computing capacity and resource constraints. The goal is to design a task scheduling algorithm that minimizes the total scheduling cost *Tct*_*i, r*_, minimizes the task completion time $t_{i,r}^{complete}$, and maximizes the resource utilization *R*_max_, all within the constraints of task completion time and budget. To achieve this, an enhanced whale optimization algorithm (EWOA) is proposed, which enhances global search capability and avoids premature convergence by introducing chaotic mapping and a nonlinear convergence factor. The algorithm optimizes task scheduling based on the whale foraging model, ultimately yielding near-optimal solutions. In summary, the primary advantages of this research are the ones that follow:

1. A multi-objective optimization model for task scheduling is devised, taking into account resource utilization, cost, and time. This model specifically tackles resource constraints on edge servers.
2. An enhanced whale optimization algorithm for task scheduling (EWOA) is proposed in this paper. To prevent task scheduling from converging into local optima and enhance the sensitivity of population initialization, a chaotic mapping model is employed. Applying chaos theory enhances sensitivity to initial conditions, resulting in a broader range of optimal scheduling strategies. Additionally, a nonlinear convergence factor is introduced to fine-tune the balance between local and global search, addressing the slow convergence issue in the traditional whale algorithm.
3. This paper establishes the experimental environment for edge computing and EWOA is contrasted with similar methods such as CATSA, WOA, HWACO, and ODTS. The numerous experiment results show that the EWOA algorithm cost is decreased by 29.22%, the average time to completion is decreased by 17.04%, and the node resource utilization is enhanced by 9.5%.

The remaining sections of the paper are structured as follows: Section 2 reviews related works. Section 3 introduces the model of the EWOA algorithm for task scheduling. Section 4 describes the design of the EWOA algorithm for task scheduling. Section 5 details the implementations of the EWOA algorithm for task scheduling. Section 6 provides an overview of the experimental environment and its configuration. Section 7 comprises the analysis and summary of the experiment. Lastly, Section 8 concludes the paper.

## 2 Related work

Task scheduling is a crucial technology in edge computing, involving the process of optimizing and transferring tasks to suitable resource repositories for execution. Extensive research has been conducted on task scheduling strategies by scholars from various countries.

To improve latency: The methodology proposed by Miao et al. (2020) first calculates the volume of data to be managed by device resource nodes, assuming each job can be divided into two sub-tasks. The study then examines the feasibility of migrating specific sub-tasks between computing nodes to reduce the latency of each job. While this approach improves system response time, its limitation is the inability to adapt to task scheduling requirements across different scenarios dynamically. He et al. (2016) proposed a particle swarm optimization algorithm for the adaptive multi-objective job scheduling methodology. This approach efficiently allocated system resources to tasks, minimizing both time and average energy consumption. However, it has the disadvantage of not being able to schedule tasks dynamically. Shrimali and Patel (2021) proposed a multi-objective optimization policy that reduces latency by efficiently managing cloud resources. While this approach enhances task execution and system responsiveness, the key challenge is balancing energy efficiency with maintaining optimal performance. To tackle the optimization challenges related to multiple objectives and workflow scheduling of tasks, Mohammadzadeh et al. (2021) presented a multi-objective optimization approach grounded in the Pareto optimum solution, which was employed to address the multi-objective scheduling problem of workflow tasks. The strength of this approach lies in the amalgamation of a greedy algorithm and a search algorithm, effectively overcoming the limitations of each and demonstrating the algorithm's advantages in high-dimensional scenarios. However, the method has drawbacks, such as high algorithmic complexity, which increases the difficulty of implementation and comprehension. Arasteh (2023) proposed a hybrid approach for software module clustering (SMC) that combines five different chaos-based metaheuristic algorithms, including Bat, Cuckoo, Teaching—Learning-Based Optimization, Black Widow, and Grasshopper algorithms. This method addresses the NP-complete problem of software module clustering by improving clustering quality and convergence speed while maintaining stability across different executions. The main advantage of this approach is its adaptability for both small and large software systems and the enhancement of clustering quality through chaos theory. In the context of task scheduling, this hybrid method could optimize the scheduling of tasks by improving the quality of task grouping, minimizing inter-task dependencies, and ensuring efficient resource allocation, thus enhancing overall scheduling performance and reducing execution time.

In order to increase resource utilization: Keshanchi et al. (2017) introduced a job scheduling method dependent on a genetic program, utilizing a heterogeneous earliest completion time search. However, a drawback of this method is the extended time required for solution detection. Sanaj and Prathap (2021) presented a hybrid job scheduling algorithm for cloud computing that combines Ant Colony Optimization (ACO) and whale optimization algorithm (WOA). Experimental results indicate the algorithm actually outperforms ACO and WOA algorithms in cloud rescheduling. However, the study did not address key aspects such as energy consumption and fault tolerance, which are critical in real-time cloud operations. A load-balancing approach aimed at maximizing revenue from edge computing was introduced by Ma et al. (2021). The approach involves allocating computational resources with the highest number of available cores and the lowest energy consumption to newly arrived tasks. However, the study assumes negligible data transmission times due to 5G networks and does not fully explore the impact of transmission delays, which could affect performance in larger-scale MEC deployments. Singh et al. (2021) proposed the QRAS (QoS-based Resource Allocation and Scheduling) algorithm for efficient task scheduling in cloud computing environments. This approach leverages Ant Colony Optimization (ACO) to optimize cloud resource allocation, improving execution cost and resource utilization. However, its emphasis on minimizing execution costs and resource use does not fully address energy consumption or the adaptability of the approach in dynamic, real-time cloud environments. Arasteh et al. (2022) outlined a method combining Grey Wolf Optimization (GWO) and Genetic Algorithm (GA) for software module clustering. It enhances modularization quality by increasing cohesion and reducing coupling, offering faster convergence and higher success rates. This method can also improve resource utilization in task scheduling by optimizing task grouping and minimizing dependencies, leading to more efficient resource allocation and scheduling.

Task Scheduling Strategies: Jing et al. (2021) provided a scheduling algorithm for QoS-aware, and the exploration results indicate that the algorithm can effectively enhance performance and achieve high reliability. While it excels in reliability, there is room for further optimization in task completion time. Rathore et al. (2020) employed the WOA and Huffman coding method for wireless sensor networks, offering innovative approaches to resource scheduling in cloud computing. However, a key limitation of the study is that it assumes an idealized underwater environment, neglecting potential real-world factors such as sensor node failures or varying water currents. Chen et al. (2021) suggested a particle swarm optimization algorithm (PSO) utilizing crossover and mutation operators to facilitate population renewal. The approach optimizes the offloading of DNN layers to reduce system energy consumption while meeting deadline constraints. However, the study assumes stable cloud-edge environments, which may not fully capture the dynamic and unpredictable nature of real-world networks. Wang et al. (2021) proposed a dependent task offloading scheme for edge computing environments based on Deep Reinforcement Learning (DRL). Their approach models the task offloading problem as a Markov Decision Process (MDP), taking into account the intrinsic dependencies among tasks represented by a Directed Acyclic Graph (DAG). Experimental results demonstrate that the DRL-based solution reduces both latency and energy consumption, outperforming heuristic and other learning-based methods. However, the study does not fully address potential scalability challenges in larger, more complex networks. Luo et al. (2020) employed an immediate time edge scheduling approach based on order-level demand. This approach considers both real-time order insertion and arrival order insertion, ensuring the fulfillment of personalized customer demands. The model demonstrates strong performance in shop floor-level metrics, including completion time, energy consumption, and resource utilization. Wen et al. (2020) outlined task scheduling based on Software-Defined Networking (SDN). Data centers based on SDN can flexibly schedule user requests without relying on custom load-balancing devices. Load balancing was achieved with a scalable minimum connection approach, addressing scheduling issues in industrial workflow applications. Zhang et al. (2019) presented the Decentralized Multi-Service Provider Resource Allocation (DMRA) scheme for resource allocation in multi-SP Mobile Edge Computing (MEC) environments. The goal of DMRA is to maximize the total profit of all service providers (SPs) while ensuring efficient resource utilization in a densely deployed network. The proposed algorithm addresses the resource allocation problem by transforming it into a user equipment (UE) and base station (BS) matching problem, achieving better service quality and profit for SPs compared to other existing methods. However, one limitation is that the study does not consider the impact of real-world network variations, such as dynamic changes in user demands or network conditions, which could affect the scalability and adaptability of the proposed solution in practical deployments. Zade et al. (2021) introduced the SAEA (Security-Aware and Energy-Aware) task scheduling strategy for cloud computing environments, utilizing the Parallel Squirrel Search Algorithm (PSSA). This approach addresses the trade-off between security and energy consumption while optimizing performance metrics such as makespan and execution time. However, the study primarily focuses on static cloud environments and does not fully account for the dynamic nature of real-time cloud systems, where rapid fluctuations in resource demands may impact task scheduling performance. Hatami and Arasteh (2020) devised a software module clustering method based on the Ant Colony Optimization (ACO) algorithm. By utilizing ACO, this method searches a module dependency graph (MDG) of large-scale software systems to cluster interdependent modules together, addressing the NP-complete problem of software module clustering. Compared to traditional heuristic methods, this approach overcomes issues such as being prone to local optima and slow convergence. Its advantages include generating high-quality, stable clusters and achieving faster convergence. In the context of task scheduling, this method optimizes task dependencies and scheduling order, avoiding dependency conflicts during the scheduling process, improving scheduling efficiency, and finding globally optimal scheduling solutions. Arasteh et al. (2023) proposed a discretized Sand Cat Swarm Optimization (SCSO) algorithm for re-modularizing program source code. This method improves software module clustering by increasing cohesion, reducing coupling, and enhancing clustering quality. It offers faster convergence and better stability for NP-complete problems. This approach could also be applied to task scheduling, optimizing task grouping and resource use while minimizing dependencies for more efficient scheduling. To provide a clearer comparison of the various task scheduling approaches discussed in this section, the characteristics of each approach are summarized in Table 1.

**Table 1:** Summary of task scheduling and resource optimization methods.

**Optimization objective**	**References**	**Characteristics**
Reducing latency	Miao et al., 2020,• He et al., 2016,• Shrimali and Patel, 2021,• Mohammadzadeh et al., 2021,• Arasteh, 2023	(1) Proposed sub-task migration between nodes to reduce task latency. • (2) Developed a particle swarm optimization method for efficient multi-objective task scheduling. • (3) Suggested a multi-objective optimization policy to reduce latency by efficiently managing cloud resources. • (4) Proposed a Pareto-optimal solution for multi-objective task scheduling, combining greedy and search algorithms. • (5) Developed a hybrid method using chaos-based metaheuristics to optimize task scheduling and reduce execution time.
Increasing resource utilization	Keshanchi et al., 2017,• Sanaj and Prathap, 2021,• Ma et al., 2021,• Singh et al., 2021,• Arasteh et al., 2022	(1) Introduced a job scheduling method using a genetic program and heterogeneous earliest completion time search to optimize resource utilization. • (2) Presented a hybrid scheduling algorithm combining Ant Colony Optimization (ACO) and whale optimization algorithm (WOA) to improve cloud rescheduling performance. • (3) Proposed a load-balancing approach for maximizing revenue in edge computing by allocating computational resources with high core counts and low energy use. • (4) Developed the QRAS algorithm using ACO to optimize resource allocation in cloud computing, enhancing execution cost and resource utilization. • (5) Combined Grey Wolf Optimization (GWO) and Genetic Algorithm (GA) to improve software module clustering, increasing cohesion and reducing coupling, thus enhancing resource utilization in task scheduling.
Task scheduling strategies	Jing et al., 2021,• Rathore et al., 2020,• Chen et al., 2021,• Wang et al., 2021,• Zhang et al., 2019,• Zade et al., 2021,• Hatami and Arasteh, 2020,• Arasteh et al., 2023	(1) Provided a QoS-aware scheduling algorithm to enhance performance and reliability. • (2) Applied WOA and Huffman coding for resource scheduling in wireless sensor networks. • (3) Suggested a PSO algorithm with crossover and mutation operators to optimize DNN layer offloading, reducing energy consumption. • (4) Proposed a DRL-based task offloading scheme for edge computing, modeling dependencies with a DAG to reduce latency and energy consumption. • (5) Developed a DMRA scheme for resource allocation in MEC environments, improving service quality and profit for service providers. • (6) Introduced the SAEA task scheduling strategy for cloud computing, balancing security and energy efficiency using PSSA. • (7) Utilized ACO to solve software module clustering, optimizing task dependencies and improving scheduling efficiency. • (8) Proposed a discretized SCSO algorithm to improve software module clustering, enhancing task scheduling by optimizing task grouping and resource use.

Numerous studies have proposed scheduling task approaches, yet a significant portion of the research primarily focuses on factors like task delay or cost, often overlooking critical issues such as resource overloading at the edge server-side caused by the excessive quantity of concurrently processed data. In addition, many existing algorithms suffer from slow convergence and the risk of getting trapped in local optima, limiting their effectiveness in dynamic and high-dimensional environments. To address these challenges, this paper combines the characteristics of the server resource nodes and the user's QoS service requirements and proposes an enhanced whale optimization algorithm (EWOA) for task scheduling in edge computing environments. The EWOA improves upon traditional algorithms by introducing chaotic mapping to diversify population initialization and a nonlinear convergence factor to balance global and local search, thus preventing premature convergence and improving resource utilization and scheduling efficiency.

## 3 A model of an enhanced whale optimization algorithm for task scheduling in edge computing environments

**Figure 2:**
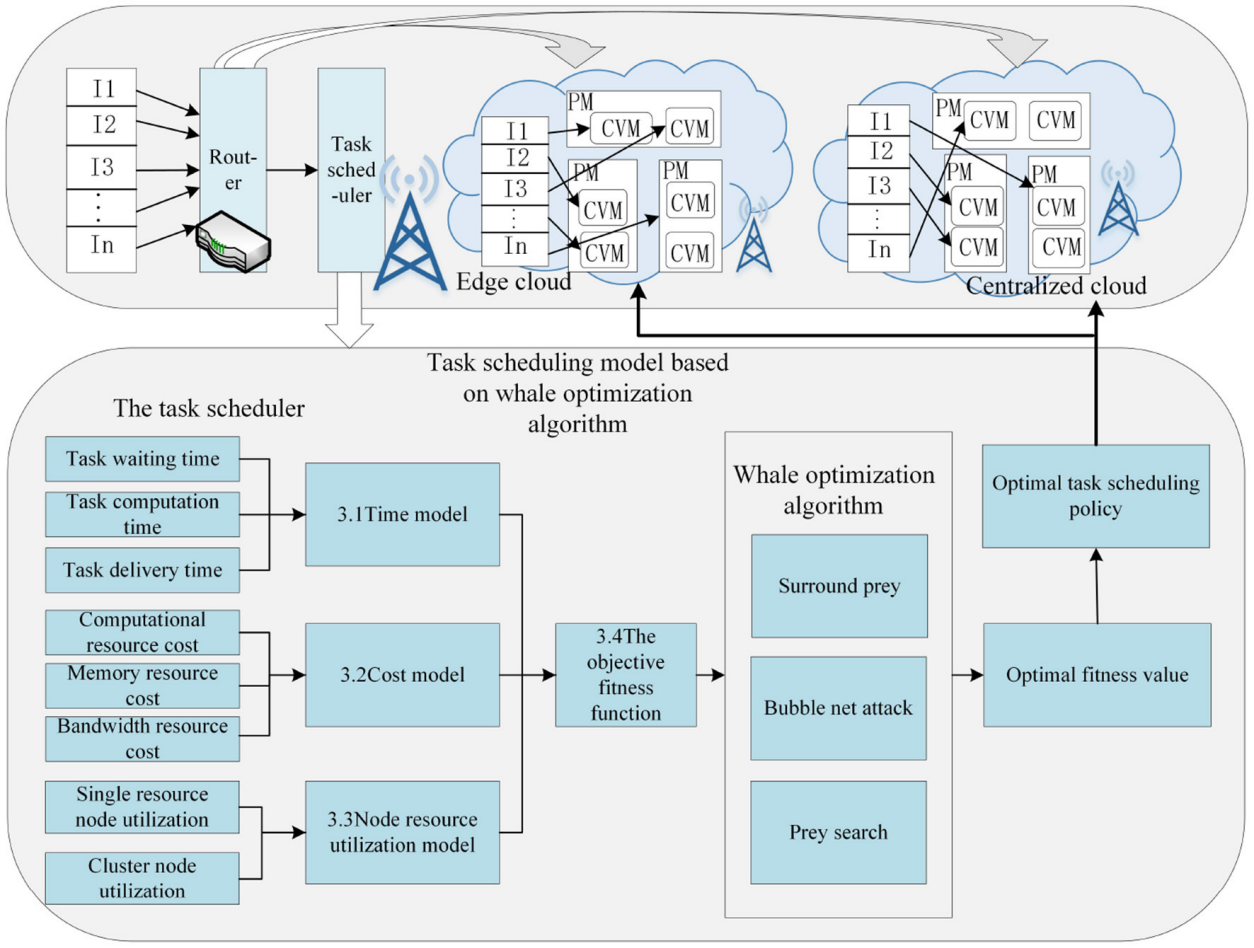
Running process of task scheduling based on enhanced whale optimization under edge computing architecture. The process evaluates tasks using the Time Model (3.1), Cost Model (3.2), and Node Resource Utilization Model (3.3). The Objective Fitness Function (3.4) determines the optimal scheduling policy, which is refined through the whale optimization algorithm's steps: Surround Prey, Bubble Net Attack, and Prey Search.

The running process of task scheduling based on enhanced whale optimization under the edge computing architecture is illustrated in Figure 2. PM indicates physical machine and VM denotes container. When users send task requests, the router will determine whether these tasks should be executed at the edge or processed in the centralized cloud. It will allocate all *i* tasks to *r* container nodes in a reasonable manner. The router will construct the target fitness function grounded in the nodes' task resource utilization, time, and cost model. Subsequently, it employs the three steps of the EWOA algorithm, which include prey search, bubble net attack, and surrounding the prey, to determine the optimal fitness value and the optimal task scheduling strategy.

The edge computing system environment is depicted as a data center, or DC, composed of a group of physical computers represented as *M* = {*M*_1_, *M*_2_, *M*_3_, ⋯ , *M*_*m*_}. These physical resources utilize virtualization technology, allowing multiple virtual computers to be deployed on a single physical machine to maximize resource utilization and reduce device consumption. Therefore, it is presumed that the virtual machines are the compute nodes of the edge cloud data center. The physical computer resource M is represented by a group of resource nodes labeled as *R* = {*R*_1_, *R*_2_, *R*_3_, ⋯ , *R*_*r*_} and a collection of tasks designated as *I* = {*I*_1_, *I*_2_, *I*_3_⋯*I*_*i*_}, *i* > *r*. The scheduling of tasks beneath the edge cloud system for computing can be expressed with the matrix that follows *T* using Equation 1, where *T*_*i, r*_ is an option variable and *T*_*i, r*_ = 1 implies which the *i*-th job is executed on the *r*-th resource node. and the implication of the primary parameters employed in this research is displayed in Table 2.


(1) $$\begin{array}{l} T=\left[\begin{array}{cccc} T_{11} & T_{12} & \cdots & T_{1r}\\ T_{21} & T_{22} & \cdots & T_{2r}\\ \cdots & \cdots & \ddots & \cdots \\ T_{i1} & T_{i2} & \cdots & T_{ir} \end{array}\right] \end{array}$$

**Table 2:** Parameter representation.

**Parameters**	**Definition**
*R*	Virtual resource node set/number
*r*	MEC Server Resource Index *r*∈*R*
*I*	Task set
*T*_*i, r*_	Task *i* is processed on resource node *r*
$t_{i,r}^{complete}$	Task *i* completion time on resource node *r*
$t_{i,r}^{wait}$	Task *i* preparation time on resource node *r*
${\text{t}}_{i,r}^{comp}$	Task *i* processing time on resource node *r*
$t_{i,r}^{send}$	Task *i* transmission time on resource node *r*
*PT*_*i, r*_	Task *i* processing time on MEC node *r*
*inst*	Total number of instructions for the task
*mips*_*r*_	Number of instructions for processing jobs per unit time for resource node *r*
*D*_*i, r*_	Data size of task *T*_*i, r*_
*ds*_*r*_	Disk speed of resource node *r*
*AFT*_*i, r*_	Actual time for task *i* to complete on MEC server *r*
*RT*_*i, r*_	Lead time for Task *i*
*DL*_*i*_	Deadline for user request assignment requirements
*pre*(*T*_*i, r*_)	The set of direct predecessors of task *T*_*i, r*_
*AT*_*i, r*_	The earliest time available for task *i* on MEC resource node *r*
*EST*_*i, r*_	Earliest beginning time of job *T*_*i, r*_ on MEC resource node *r*
*ub*_*i*_	Expected cost of task A implementation *i*
*Tct*_*i, r*_	The total cost necessary for job *i* to be accomplished on resource node *r*
*Cct*_*i, r*_	Computed resource cost of job *i* on resource node *r*
*Sct*_*i, r*_	Cost of storage resources for task *i* on resource node *r*
*Bct*_*i, r*_	Transmission bandwidth for the resource node *r* executing task *i*
α	Indicates the unit service price for task calculation
β	Unit Service Price for Bandwidth Allocation
γ	Unit service price for storage allocation
*P*_*r*_	Computing power of server nodes
*R*_*node*_	Node Utilization
*R*_*cluster*_	Cluster Utilization
*R*_max_	Maximum resource utilization

### 3.1 Time model

The edge router assumes a pivotal role in determining whether a task should be executed at the edge or transmitted to the cloud. This decision is contingent upon the constrained computing power of each physical machine, thereby influencing the allocation of jobs to either the cloud or the edge. When jobs are scheduled to the cloud or edge, the task completion time $t_{i,r}^{complete}$ encompasses the sum of preparation time $t_{i,r}^{wait}$, processing time ${\text{t}}_{i,r}^{comp}$, and task transfer time $t_{i,r}^{send}$. The processing time is further composed of the computation time spent on a node and the data processing time on the disk. *PT*_*i, r*_ represents the task *T*_*i, r*_ processing time on the edge server *r*, Which can be expressed as follows:


(2) $$\begin{array}{l} t_{i,r}^{comp}=PT_{i,r}=\frac{inst}{mips_{r}}+\frac{D_{i,r}}{ds_{r}} \end{array}$$

In Equation 2, *inst* represents the aggregate amount of task instructions. *mips*_*r*_ stands for the amount of instructions for node *r* to carry out tasks within a given period. *D*_*i, r*_ denotes the data size of task *T*_*i, r*_. *ds*_*r*_ stands for the disk speed of node *r*.

The time of task transmission to the node can be defined as:


(3) $$\begin{array}{l} t_{i,r}^{send}=\frac{D_{i,r}}{B\log_{2}\left(1+\frac{P_{u}Los}{\sigma }\right)} \end{array}$$

In Equation 3, *Los* is the channel power gain. *P*_*u*_ indicates the transmit power. *B* denotes the bandwidth. σ stands for the Gaussian noise power in the channel. The level of service is defined as a distance-based function *Los* = *d*^−α^ based on a model of wireless interference in a cellular wireless environment, according to the literature (Rappaport, 2002), the value of α is 4. In the calculation, σ is defined as −100*dB*_*m*_.

It is expected that every MEC node on the RSU can just perform one job each time; hence, subsequent duties issued to the identical MEC resource node are unlikely to be completed promptly since current tasks are being handled on the similar MEC resource node. *AFT*_*i, r*_ is denoted as the actual moment when job *T*_*i, r*_ is finished on the MEC resource node, *T*_*i, r*_represents the *i*-th task of application *T*_*m*_ that is processed on resource node *r*, and a task's ready time is the earliest time when all its immediate antecedents are completed. Thus, the preparation time *RT*_*i, r*_ of task *T*_*i, r*_ can be calculated as:


(4) $$\begin{array}{l} RT_{i,r}=\underset{T_{i=1,r}\in pre\left(T_{i,r}\right)}{\max}AFT_{i-1,r} \end{array}$$

In Equation 4, *pre*(*T*_*i*−1, *r*_) indicates the set of immediate antecedents of task *T*_*i, r*_. *T*_*i*−1, *r*_ must be completed before task *T*_*i, r*_ can be begun.

In addition, assuming that *r*∈*R* represents a MEC resource node on an RSU when MEC resource node *r* is idle, task *T*_*i, r*_ can be scheduled on the MEC resource node *r*. If another job is executing on MEC resource node *r*, then the task has to remain in line while MEC resource node *r* is accessible. *AT*_*i, r*_ denotes the earliest time available for a task *i* on MEC resource node *r*. A job can be launched using a MEC resource node when it is ready and a MEC resource node is available for that task.

The earliest starting time for a job occurs once the job is prepared, and the MEC resource node becomes available for that job. Therefore, the earliest starting time *EST*_*i, r*_ for job *T*_*i, r*_ on the MEC resource node can be calculated as follows:


(5) $$\begin{array}{l} t_{i,r}^{wait}=EST_{i,r}=\max\left\{RT_{i,r},AT_{i,r}\right\} \end{array}$$

Thus, the total task completion time $t_{i,r}^{complete}$ can be defined as:


(6) $$\begin{array}{l} \overset{I}{\underset{i=1}{\sum }}\overset{R}{\underset{r=1}{\sum }}t_{i,r}^{complete}=t_{i,r}^{wait}+t_{i,r}^{comp}+t_{i,r}^{send} \end{array}$$

Furthermore, the task completion time must be within the deadline *DL*_*i*_ set by the user to meet the task requirements. If $t_{i,r}^{complete}\leq DL_{i}$ is true, the task can be executed on the edge clouds. Otherwise, the task will be scheduled on the centralized cloud. The centralized cloud guarantees task completion due to its extensive computational resources and scalability, efficiently handling high volumes of tasks. However, the architecture also incorporates edge clouds to minimize latency, enhance real-time processing, and alleviate the load on the centralized cloud. By processing tasks closer to the data source, edge clouds improve response times and optimize resource utilization, which is crucial for scenarios requiring immediate processing.

### 3.2 Cost model

The total cost of centralized task processing needs to be estimated, regardless of whether the task is scheduled to be performed at the edge or in the centralized cloud. The total cost *Tct*_*i, r*_ includes the *Cct*_*i, r*_ compute resource cost, the *Sct*_*i, r*_ storage resource cost, and the *Bct*_*i, r*_ bandwidth resource cost. The total cost can be composed as:


(7) $$\begin{array}{l} Tct_{i,r}=\overset{I}{\underset{i=1}{\sum }}\overset{R}{\underset{r=1}{\sum }}\left(Cct_{i,r}+Sct_{i,r}+Bct_{i,r}\right) \end{array}$$

where the compute resource cost can be represented as follows:


(8) $$\begin{array}{l} Cct_{i,r}=\left[\frac{inst}{mips_{r}}+\frac{D_{i,r}}{ds_{r}}\right]\times \alpha \end{array}$$

In Equation 8, *inst* is the total task instructions count. *mips*_*r*_ indicates the count of directives to be processed per unit of time at server node *r*. *ds*_*r*_ means the disk speed of node *r*. *D*_*i, r*_ denotes the data size of task *T*_*i, r*_. α represents the price per unit of service for task computation.

The cost of storage resources can be stated as:


(9) $$\begin{array}{l} Sct_{i,r}=stor_{i,r}\times \beta \end{array}$$

In Equation 9, *stor*_*i, r*_ indicates the storage space for server resource node *r* to allocate task *i*. β represents the price per unit of service for storage allocation.

Bandwidth resource costs can be expressed as follows:


(10) $$\begin{array}{l} Bct_{i,r}=band_{i,r}\times \gamma \end{array}$$

In Equation 10, *Bct*_*i, r*_ is the communication bandwidth for resource *r* to execute task *i*. γ represents the price per unit of service for bandwidth allocation.

Therefore, when the total cost *Tct*_*i, r*_ is less than the budgeted cost *ub*_*i*_ for the user to send task *i*, then *Tct*_*i, r*_ ≤ *ub*_*i*_, and the task is able to be taken on the consolidated cloud.

### 3.3 Resource utilization model

When tasks are scheduled to the edge cloud, the edge orchestrator monitors the system by tracking resource utilization, task completion times, and overall system performance. This continuous monitoring facilitates dynamic adjustments in resource allocation and task scheduling, ensuring cluster load balance and minimizing execution time. The compute power *P*_*r*_ of the edge server can be phrased as:


(11) $$\begin{array}{l} P_{r}=\omega_{1}\frac{P_{cpu}}{\min\left(P_{cpu}\right)}+\omega_{2}\frac{P_{ram}}{\min\left(P_{ram}\right)} \end{array}$$

In Equation 11, the edge server's CPU efficiency and memory performance are denoted by *P*_*cpu*_ and *P*_*ram*_, respectively. ω_1_ and ω_2_ are the weighting factors, and ω_1_+ω_2_ = 1. If the task demands more CPU capabilities than memory resources, ω_1_ is specified to be greater than ω_2_.

In addition, the node's utilization rate *R*_*node*_ can be expressed as:


(12) $$\begin{array}{l} R_{node}=100\%\times U_{node}/S_{node} \end{array}$$

In Equation 12, *S*_*node*_ is the node storage and *U*_*node*_ is the storage used by users to send task requests.

Thus, cluster utilization *R*_*cluster*_ is described as:


(13) $$\begin{array}{l} R_{cluster}\left(r\right)=100\%\times U_{cluster}\left(r\right)/S_{cluter}\left(r\right)=100\%\\ \times \sum_{r=1}^{N_{node}}U_{node}\left(r\right)/\sum_{r=1}^{N_{node}}S_{node}\left(r\right) \end{array}$$

To address the potential impact of excess load on cluster performance and task failure, this paper introduces a threshold value λ. If the cluster load surpasses this threshold, the edge scheduler suspends the assignment of tasks to the edge servers. Additionally, it is essential for node utilization to remain below the defined maximum *R*_max_. The maximal resource utilization *R*_max_ is defined as:


(14) $$\begin{array}{l} R_{\max}=\left[\lambda +\left(1-\lambda \right)\times R_{cluster}\right]\times 100\% \end{array}$$

The objective function for task scheduling can be rendered as:


(15) $$\begin{array}{l} \min\left\{\overset{I}{\underset{i=1}{\sum }}\overset{R}{\underset{r=1}{\sum }}\left[t_{i,r}^{complete}+Tct_{i,r}\right]\right\} \end{array}$$

(16) $$\begin{array}{l} \max R_{\max} \end{array}$$

(17) $$\begin{array}{l} s.t.\left\{ \begin{array}{c} t_{i,r}^{complete}\leq DL_{i,r}\\ Tct_{i,r}\leq ub_{i,r}\\ R_{node}\leq R_{\max} \end{array}\right. \end{array}$$

### 3.4 Constructing the fitness function

The multi-objective model assesses the cost function, taking into account the CPU and memory of the central processor. The fitness function is then determined by incorporating the completion time, cost function, and resource utilization of the service nodes. Based on the obtained adaptation values, tasks are assigned to cloud or edge resource nodes employing the optimal strategy. The fitness function is rendered as:


(18) $$\begin{array}{c} \min Fit=\\ \lambda_{1}\min\overset{I}{\underset{i=1}{\sum }}\overset{R}{\underset{r=1}{\sum }}t_{i,r}^{complete}+\lambda_{2}\min\overset{I}{\underset{i=1}{\sum }}\overset{R}{\underset{r=1}{\sum }}Tct_{i,r}+\lambda_{3}\min\frac{1}{\max R_{\max}} \end{array}$$

In Equation 18, λ_1_ ∈ [0, 1], λ_2_ ∈ [0, 1], λ_3_ ∈ [0, 1], and λ_1_+λ_2_+λ_3_ = 1. It is evident that minimizing of task resource utilization, execution cost, and completion time [i.e., a smaller *Fit*(*x*)] leads to better optimization performance. Conversely, a larger *Fit*(*x*) is associated with poorer optimization performance.

## 4 Design of an enhanced whale optimization algorithm for task scheduling in edge computing environments

**Figure 3:**
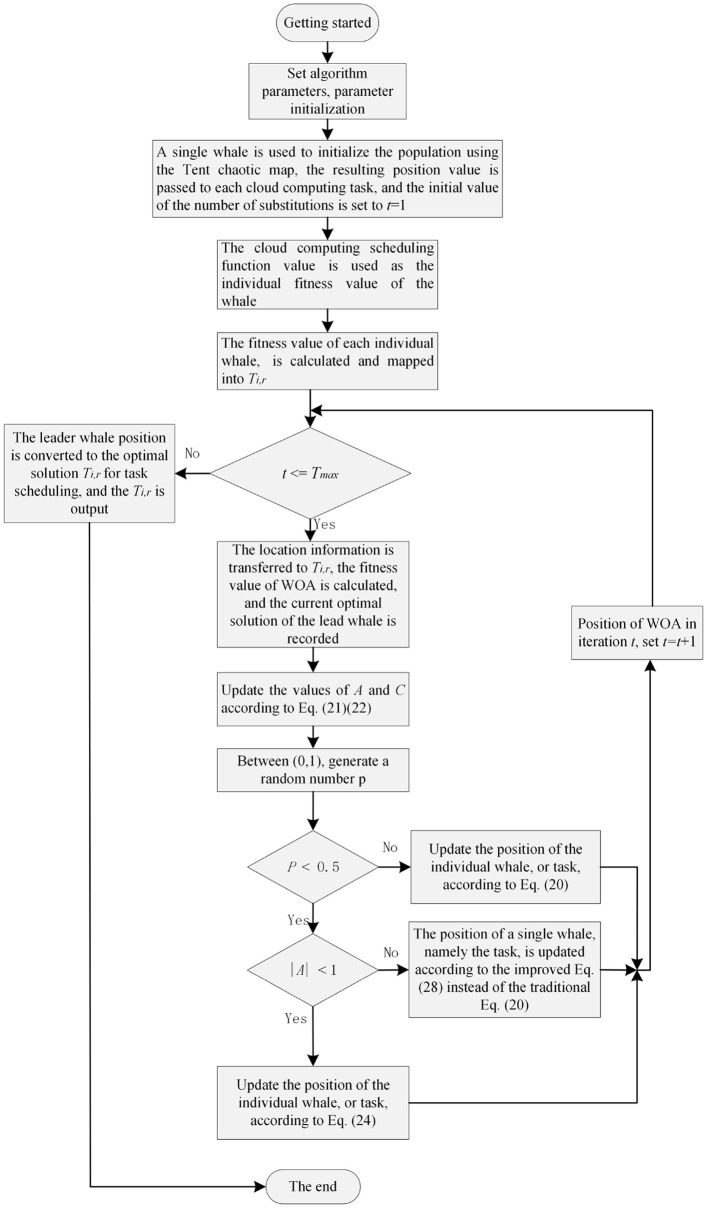
Based on the enhanced whale optimization algorithm process.

This section presents the task scheduling method based on the enhanced whale optimization algorithm (EWOA), which employs a multi-objective optimization approach utilizing time, cost, and resource utilization models. Each model calculates the task completion time, execution cost, and resource utilization of the nodes. The fitness function is then used to assess the effectiveness of the scheduling solutions. The whale optimization algorithm optimizes task allocation through three key steps: Surround Prey, Bubble Net Attack, and Prey Search, progressively refining the task distribution. The final outcome is an optimal scheduling policy that maximizes resource utilization while minimizing both cost and time. The task scheduling flow based on the EWOA algorithm is shown in Figure 3. The following subsections will detail each step.

### 4.1 Task scheduling algorithm description

Throughout the search process, the position *X* of the whale is encoded as a vector of length *N*, where the whale's position corresponds to the solution *T*_*i, r*_ of the scheduling problem, with the lead whale representing the current optimal solution. The leading whale's fitness function value indicates the optimal solution for *Fit*. In this scenario, the WOA algorithm can be employed to discover a more efficient approach for scheduling jobs in edge cloud computing systems. That is, in *t* iterations of the WOA, as all whales renew their positions, each whale's positional information is transmitted to the solution *T*_*i, r*_ of the fixed task. The whale fitness function denotes the value of *Fit*(*x*). Using the values in matrix *T*_*i, r*_, calculate the fitness value *Fit* for the whale. The lead whale will be the whale with the minimum value and will use its position information to update the positions of the other whales in the next iteration. Repeat these processes until the optimal scheduling scheme is achieved. Eventually, the lead whale's location information will be transmitted to *T*_*i, r*_. This solution will generate a scheduling plan for optimizing tasks edge cloud computing system. In the WOA algorithm, it is generally divided into three distinct phases:

1. Encircling prey
  The purpose of the current phase is for a task to search for container VMs within a specific area. At the initial stage of the algorithm, the humpback whale symbolizes the optimal solution between the task and the virtual resource node, and the food represents the container VM. When tasks are unaware of the locations of virtual resources, they can collaborate in groups to discover the location of container VMs. Thus, the whale nearest to the virtual machine equals the present local optimum approach. The single whale represents the task, and others can approach this spot and gradually encircle their prey. It is able to be displayed using the below mathematical model:
(19) $$\begin{array}{l} \vec{D}=|C\times \vec{X^{*}}\left(t\right)-\vec{X}\left(t\right)| \end{array}$$
(20) $$\begin{array}{l} \vec{X}\left(t+1\right)=\vec{X^{*}}\left(t\right)-A\times \vec{D} \end{array}$$
  In Equations 19, 20, where $\vec{D}$ represents the vector of distances from the searching agent to the objective food. $\vec{X^{*}}\left(t\right)$ signifies the humpback whale's present value, $\vec{X}\left(t\right)$ symbolizes the vector of location. *t* denotes the current iteration amount. *C* and *A* are vector coefficients that can be symbolized as:
(21) $$\begin{array}{l} C=2\times d \end{array}$$
(22) $$\begin{array}{l} A=2\times a\times d-a \end{array}$$
(23) $$\begin{array}{l} a=2-\frac{2t}{T_{\max}} \end{array}$$
  In Equations 19–21, *a* signifies a straight reduction from 2 to 0, and *d* is a randomly chosen number between 0 and 1. *a* represents a linear value based on the maximum iteration times *T*_max_ with the iteration times *t* decreasing from 2 to 0.

2. Bubble-net attack
  The objective of the current phase is to simulate humpback whales executing bubble attacks. This involves designing the whales' feeding and bubbling behaviors by narrowing the surround and updating the spiral position to achieve the whale's local optimization. Then the behavior of the whales' bubble net attack is modeled. This phase is divided into two steps:
  1) Shrinking envelope: From Equation 22, it can be seen that when |*A*| < 1, the whale will shrink the envelope. That is, the individual whale will approach the prey at its present optimal position and swim around it in a progressively shrinking circle. The bigger the value of |*A*|, the greater the steps the whale will take, and the reverse is true.
  2) Spiral position update: Every humpback whale initially computes its proximity to the present best whale, and subsequently shifts along the spiral path. The location renew-al process can be expressed as:
(24) $$\begin{array}{l} \vec{X}\left(t+1\right)={\vec{D}}^{\prime }\times e^{lb}\times \cos\left(2\pi l\right)+\vec{X}*\left(t\right) \end{array}$$
  In Equation 24, $\vec{D}=|\vec{X}*\left(t\right)-\vec{X}\left(t\right)|$ represents a vector, being the away from a single whale to the finest whale (presently finest find), i.e., the separation between the virtual machine and *i*-th task (presently optimal solution). *l* signifies a randomly selected number ranging from -1 to 1, and *b* signifies a constant.
  To model both behaviors simultaneously, postulate that the whale updating probability of its position according to the spiraled path and the contraction path is set at 0.5 respectively, and can be represented as:
(25) $$\begin{array}{l} \vec{X}\left(t+1\right)=\left\{ \begin{array}{ll} {\vec{X}}^{*}\left(t\right)-\vec{D}\times A & p<0.5,\\ e^{lb}\times \vec{D}\times \cos\left(2\pi l\right)+{\vec{X}}^{*}\left(t\right) & p\geq 0.5. \end{array}\right. \end{array}$$
  Where *p* designates an arbitrarily produced value between 0 and 1.

3. Prey search The goal is to ensure that the fitness function attains an approximately globally optimal solution. When |*A*|>1, search agents repel each other. In this scenario, a randomly chosen search agent will supplant the present optimal search agent location. The corresponding model mathematically can be stated as:
(26) $$\begin{array}{l} \left\{ \begin{array}{l} \vec{D}=|C\times {\vec{X}}_{\text{rand}}\left(t\right)-\vec{X}\left(t\right)|\\ \vec{X}\left(t+1\right)={\vec{X}}_{\text{rand}}-A\times |C\times {\vec{X}}_{\text{rand}}-\vec{X}\left(t\right)| \end{array}\right. \end{array}$$
  In Equation 26, ${\vec{X}}_{rand}$ stands for a random select vector location of a search agent, signifying a randomized task.

### 4.2 Task scheduling algorithm based on enhanced whale optimization

The enhanced whale optimization algorithm (EWOA) builds upon the standard WOA by incorporating chaotic mapping to prevent premature convergence and ensure population diversity, along with a nonlinear convergence factor to balance local and global search. These modifications enhance the algorithm's ability to efficiently allocate tasks in resource-constrained edge computing environments, where dynamic network conditions and limited resources require real-time, adaptive task scheduling.

#### 4.2.1 Chaotic mapping

Most population algorithms commonly employ Gaussian or uniform distributions for initializing individuals. However, this approach produces an unresponsive initial population. Chaos theory is able to render the initial circumstances more responsive and generate a more changeable range of numbers (Sayed et al., 2018). Consequently, numerous specialists and researchers Gupta and Deep (2019) and Li et al. (2018) have concentrated on devising diverse chaotic mappings to circumvent random population initialization. The random initialization employed by the whale optimization algorithm in population initialization can easily lead to uneven spatial distribution and susceptibility to falling into local optima (Kaur and Arora, 2018). Diverse population initialization values can be achieved by employing distinct mapping functions, ensuring uniform distribution, and broadening the search span of whale initialization. By integrating a chaos mechanism (Teng et al., 2019; Zhu et al., 2020), algorithmic randomness can be minimized, preventing premature convergence and enhancing overall performance. In this paper, Tent chaotic mapping is employed to generate randomized chaotic sequences, thereby constituting an initial population of whales and enhancing the algorithm's global search capability. The specific chaos mapping can be shown as:


(27) $$\begin{array}{l} w\left(t+1\right)=\left\{ \begin{array}{ll} 2w\left(t\right), & 0<w\left(t\right)<0.5,\\ 2\left(1-w\left(t\right)\right), & 0.5\leq w\left(t\right)<1. \end{array}\right. \end{array}$$

The improved position update, i.e., Equation 20 is improved as:


(28) $$\begin{array}{l} X\left(t+1\right)=w\left(t\right)\cdot X_{rand}-A\cdot D \end{array}$$

#### 4.2.2 Non-linear convergence factor

As evident from the fundamental whale algorithm, when |*A*|≥1, to find better-targeted prey the whale begins to enlarge the search zone; When |*A*| < 1, it contracts the envelope and approaches the prey, thereby enhancing the localization of the algorithm. When this behavior is translated into a mathematical model, it becomes apparent that the vector *A* is the crucial factor determining the degree of optimization and the convergence speed. From Equation 22, the most important variable influencing the vector is *a*. From Equations 21–23, the value of *a* determines the *a* fluctuations range. *a* can fluctuate linearly with iterations amount, however, the relational equation of *a* includes random values. Besides *A*, the location update Equation 26 for WOA includes a randomly selected search agent and the coefficient *C* is dependent on the random values. A mass of random number values causes the algorithm to become excessively random, which reduces the convergent speed and the algorithm's precision. A small number of random values leads to the algorithm cluster close to the optimum value throughout iterations, thus prematurely stopping convergence and falling into the local optimum. Thus, the second enhancement point is improving the linearly decreasing quantity.

The objective of this paper is to boost the whale's pre-search capability by optimizing the algorithm's convergence rate, achieving a harmonious balance between the local exploitation capabilities and the global search of WOA. Equation 23 is expressed after improving *a* as:


(29) $$\begin{array}{l} a=a_{top}-a_{bottom}\times \frac{T_{\max}-t}{T_{\max}} \end{array}$$

In Equation 29, *a*_*bottom*_ represents the final value of *a*. The initial value of *a* is denoted by *a*_*top*_. *T*_max_ indicates the utmost iteration number, and *t* stands for the current iteration count. Given that whales exhibit nonlinearity during the detection phase, a nonlinear convergence factor has been incorporated to enhance the equilibrium between the algorithm's local search abilities and global search abilities.

## 5 Implementation of an enhanced whale optimization algorithm for task scheduling in edge computing environments

### 5.1 Implementation of an enhanced whale optimization algorithm for task scheduling

The enhanced whale optimization algorithm (EWOA) is designed to handle task scheduling in dynamic, resource-constrained environments such as edge computing. The algorithm optimizes task allocation through the key steps of Surround Prey, Bubble Net Attack, and Prey Search, ensuring tasks are distributed efficiently across available nodes. Computations are primarily handled by edge servers, which execute the tasks based on real-time resource availability and system load. EWOA dynamically assigns tasks to these edge servers by considering resource utilization, task completion time, and execution cost. If edge resources become limited, tasks may be offloaded to the local cloud to prevent overload and ensure timely completion. This dynamic task allocation mechanism ensures that the workload is balanced across nodes and optimally distributed.

The EWOA begins by formulating the objective function for task scheduling, taking into account node resource utilization, cost, and task completion time. Following this, it defines the fitness function in accordance with the target function, ultimately pinpointing the Optimal strategy for task scheduling through the EWOA algorithm. Refer to Algorithm 1 for the detailed pseudocode.

**Algorithm 1:**
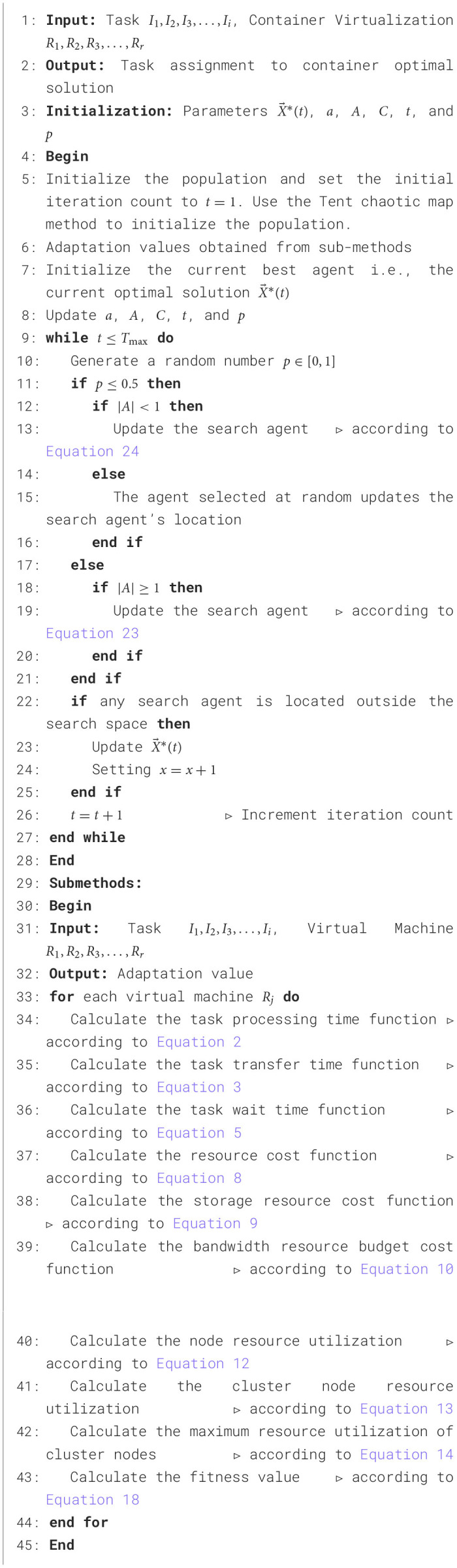
An enhanced whale optimization algorithm for task scheduling in edge computing environments.

### 5.2 Complexity analysis of an enhanced whale optimization algorithm for task scheduling

Time complexity is the computational effort required to execute an algorithm and depends mainly on how many times the question is repeated. In the fundamental whale optimization algorithm, time complication predominantly fluence of the search dimension (D), the number of iterations (T), and the population size (N). Therefore, in this paper, the WOA time complication denotes *O*(D*T*N). The EWOA is enhanced from the WOA by introducing the Tent chaotic mapping, which increases the amount of *O*(D*T*N) computation. So the EWOA's complexity is denoted as *O*(2D*T*N), which is upper than the standard WOA. When the optimization problem has a large spatial dimension, the time complexity of EWOA tends to be around *O*(D*T*N). Furthermore, the spatial complexity is mainly affected by population size N and the dimension of search D and search dimension D, and the spatial complexity of the two algorithms is denoted by *O*(D*N).

## 6 Experimental environment and configuration

### 6.1 Baseline algorithms

Widely recognized and commonly applied classical algorithms were selected as baselines, including ODTS (Optimal Dynamic Task Scheduling; Yuan et al., 2021), WOA (whale optimization algorithm; Hosseini et al., 2021), HWACO (Hybrid Weighted Ant Colony Optimization; Chandrashekar et al., 2023), and CATSA (Content-Aware Task Scheduling Algorithm; Lakhan and Li, 2019). These algorithms are well-defined and experimentally validated in the literature, ensuring robust representativeness. The selection of baseline algorithms considered multiple evaluation dimensions such as task scheduling time, cost, and resource utilization to comprehensively assess the algorithm's performance. Additionally, the consistency and repeatability of the baseline algorithms across different experimental environments were crucial criteria, ensuring stable execution within the experimental setup and providing reliable comparative data.

### 6.2 Environmental setting

The software environment for this experiment uses the Ubuntu-14.04.5 operating system, JKD version 1.8.0-11 development kit, the Hadoop version employed is the stable Hadoop-2.7.1, OpenVPN 2.3.2, and Linux Eclipse 4.5.0 serves as the development environment.

**Figure 4:**
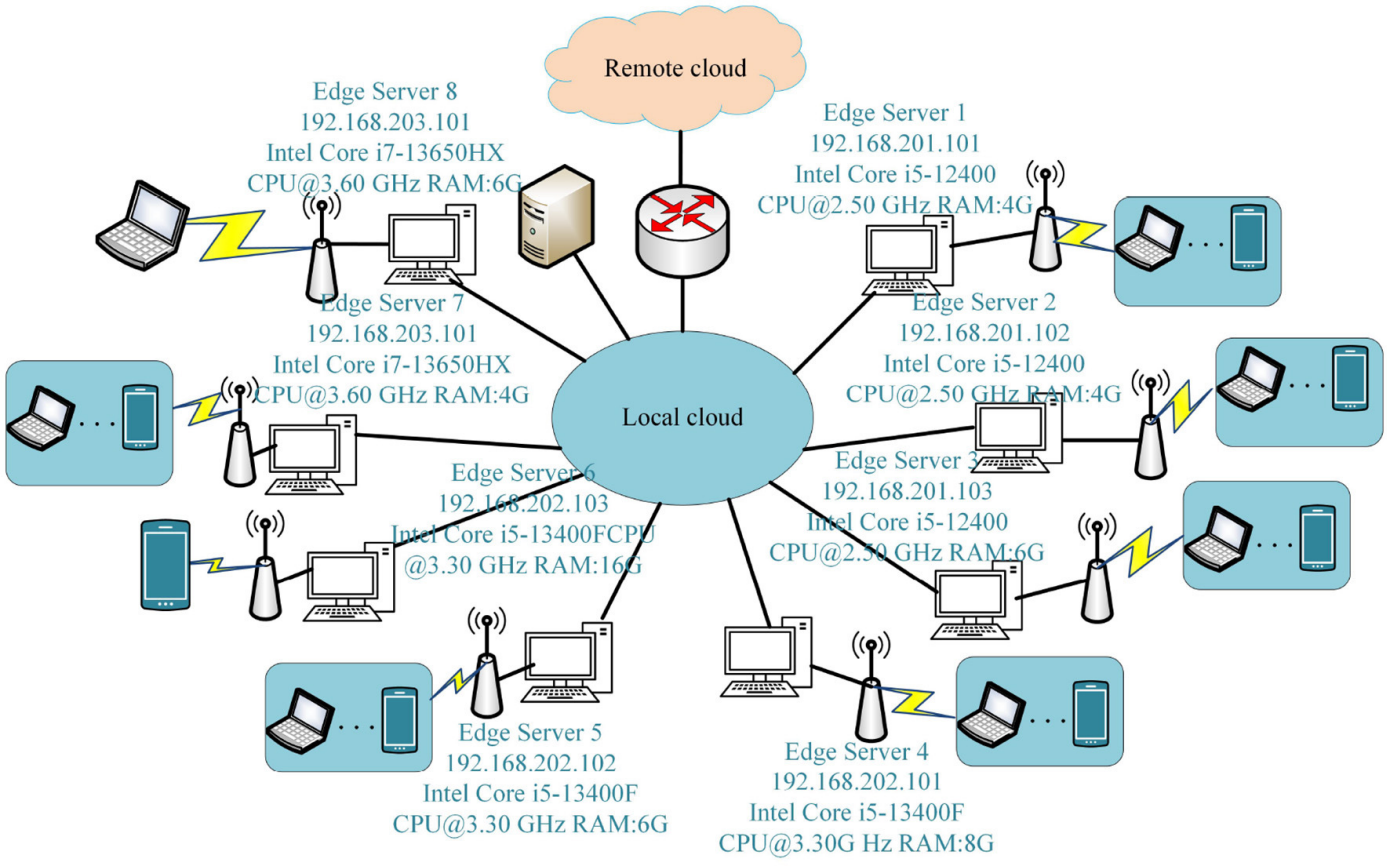
Experimental test environment.

To simulate the real scenario of the experiments in this paper, the experimental setup includes edge-end node servers and cloud servers. The hardware platform comprises three main components: edge cloud servers, cloud servers, and laptops, along with other mobile devices. In cases where the resources within the edge cloud cluster are inadequate and necessitate supplementation from the public cloud, a VPN is employed to facilitate access to the resources of the public cloud. The specific architecture of the experimental environment is illustrated in Figure 4.

During the experiment, we leveraged AliCloud's public cloud resources. Table 3 outlines the specifics of instance types, CPU specifications, and associated costs provided by the public cloud service provider for the servers. The instance cost represents the pricing for the cloud server, expressed in US dollars per second ($/s).

**Table 3:** Server instance type, instance cost, and CPU type.

**Public cloud service providers**	**Instance type**	**CPU type**	**Instance cost( × 10^−6^$/s)**
Ali cloud	Core:8 RAM:16 GB Bandwith:60 Mbps	Intel Xeon(ice lake) platinum 8369b 2.70 GHZ	35
	Core:4 RAM:8 GB Bandwith:20 Mbps		11
	Core:2 RAM:4 GB Bandwidth:40 Mbps		20

### 6.3 Test data

In this paper, the experimental data originates from the Stanford Network Analysis Project (SNAP) standard dataset (Leskovec and Krevl, 2014). This dataset compiles information from various sources such as social networking sites, online reviews, online communities, and video sites. The dataset comprises various components, including the Wikipedia network, articles, and metadata, amounting to a total data volume of 4 GB, corresponding to 31 Map tasks. User comments, questions, and answers, along with online comments related to temporal networks, constitute a data volume of 5 GB, mapped to 40 Map tasks. Additionally, Web-Flickr and wiki-Elec, with a combined data volume of 4GB, correspond to 31 Map tasks. This diverse dataset spans multiple classifications and encompasses a broad spectrum of domains, holding practical research significance.

### 6.4 Evaluation metrics

When evaluating the performance of EWOA algorithms, the considered metrics are resource utilization, cost, and completion time. These metrics are also key Quality of Service (QoS) indicators in edge computing environments, reflecting user expectations for timely and efficient task execution.

1. Completion Time: The maximum completion time signifies the total duration necessary for task execution, encompassing preparation time, task transfer time, and processing time. Minimizing completion time is crucial to meeting real-time demands and ensuring prompt service delivery, a core QoS requirement in edge computing.
2. Cost: The cost associated with scheduling a task within a container encompasses the overall expenditure on computing resources, storage resources, and bandwidth resources. Reducing execution costs is critical to maintaining cost-effective service while optimizing resource use, directly impacting user satisfaction with the service's affordability.
3. Resource Utilization: Resource utilization represents the efficiency with which resources are allocated and used for scheduling tasks to containers. Maximizing resource utilization ensures that available computational power is used effectively, preventing overuse or underuse of resources, which is vital to sustaining system performance and reliability.

By incorporating these QoS metrics into the fitness function, EWOA ensures that task scheduling aligns with user expectations for performance, cost-efficiency, and resource management.

## 7 Results and discussion

In this section, we present the results of our experiments and discuss their implications with respect to the research questions raised. The discussion is based on the findings from both small- and large-scale tasks, focusing on the performance of EWOA in terms of execution time, cost, and resource utilization compared to other algorithms.

### 7.1 Experimental results and analysis for small- and large-scale tasks

In this study, experiments are categorized into large-scale and small-scale edge computing tasks. Small-scale tasks are defined by a task count within the range [0, 100], while large-scale tasks fall within the range [100, 1,000]. The threshold of 100 was selected based on preliminary experiments, which showed significant changes in system performance and resource utilization beyond this point. Each experimental condition is repeated 20 times to ensure statistical reliability. For every algorithm, we have recorded the best, worst, and average performance metrics to provide a comprehensive evaluation of their stability and effectiveness. However, the primary focus of our analysis is on the average results, as they offer a more representative overview of overall performance.

#### 7.1.1 Analysis of experimental results under small-scale tasks

**Figure 5:**
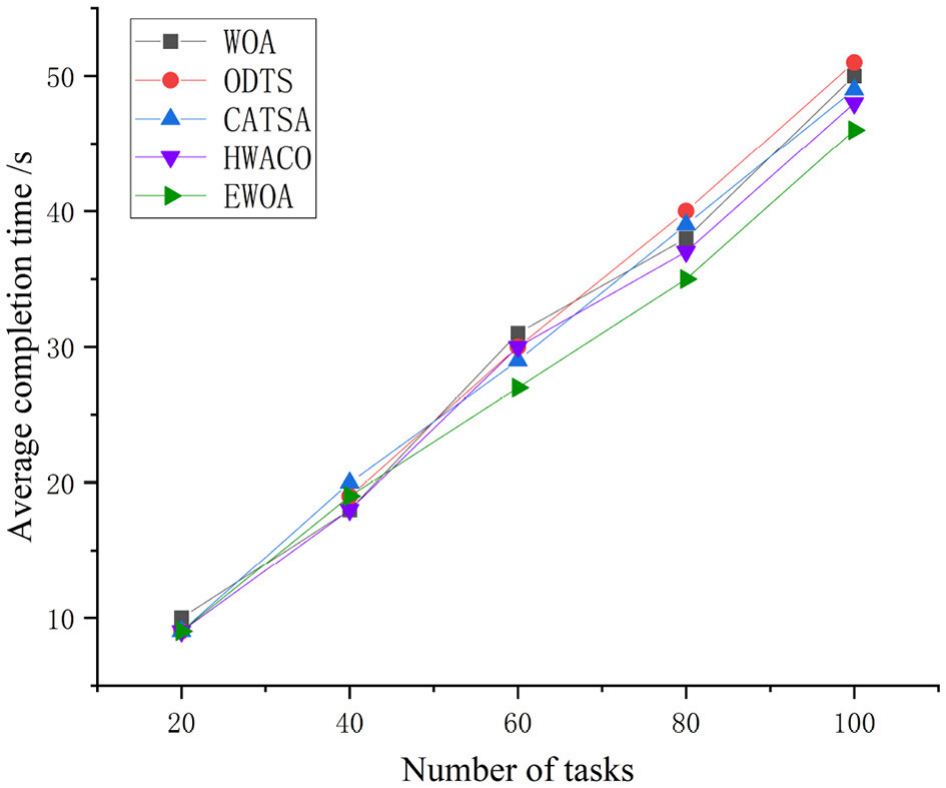
Average completion time of running the same task on a small scale.

Figure 5 clearly illustrates that with an increasing number of tasks, the completion time for all four algorithms also increases. Specifically, when the task count falls within the range [0, 30], the WOA algorithm exhibits a slower rate of increase compared to the ODTS and CATSA algorithms. Upon completing 40 tasks, the EWOA algorithm is better than three other algorithms, with its average job execution time being 7.4, 8.72, 6.84, and 4.23% lower than that of the WOA, ODTS, CATSA, and HWACO, respectively. This dis-crepancy arises from the fact that the algorithms presented in this paper take system re-source utilization into account, a factor not considered by the WOA, ODTS, and CATSA algorithms. The EWOA algorithm holds an advantage in terms of average completion time for small-scale tasks, owing to the edge scheduler's capability to monitor the state of edge servers, thereby ensuring cluster load balance and reducing execution time.

**Figure 6:**
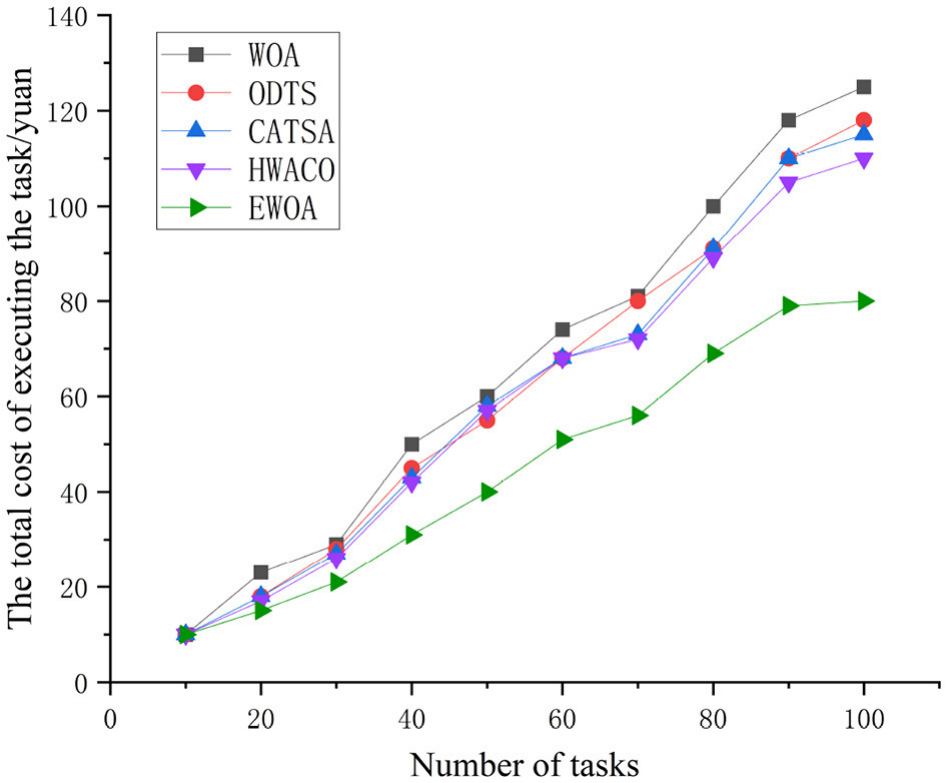
Comparison of the total cost of performing small-scale tasks.

Figure 6 illustrates that the costs associated with the WOA, ODTS, CATSA, and EWOA algorithms are nearly identical when the task count is 10. However, as the task count rises to 100, the curves for all four algorithms depict a gradual increase, demonstrating a positive correlation with the task count. In contrast, the EWOA algorithm is more cost-effective than the other three algorithms. This is attributed to the EWOA algorithm's consideration of the budget cost associated with tasks prior to their arrival. If the total cost *Tct*_*i, r*_ of executing a task is less than the budget cost *ub*_*i*_ of sending task *i*, then *Tct*_*i, r*_ ≤ *ub*_*i*_, the task can be executed on the centralized cloud. By allocating tasks correctly, the EWOA algorithm can save on the total cost of task execution.

**Figure 7:**
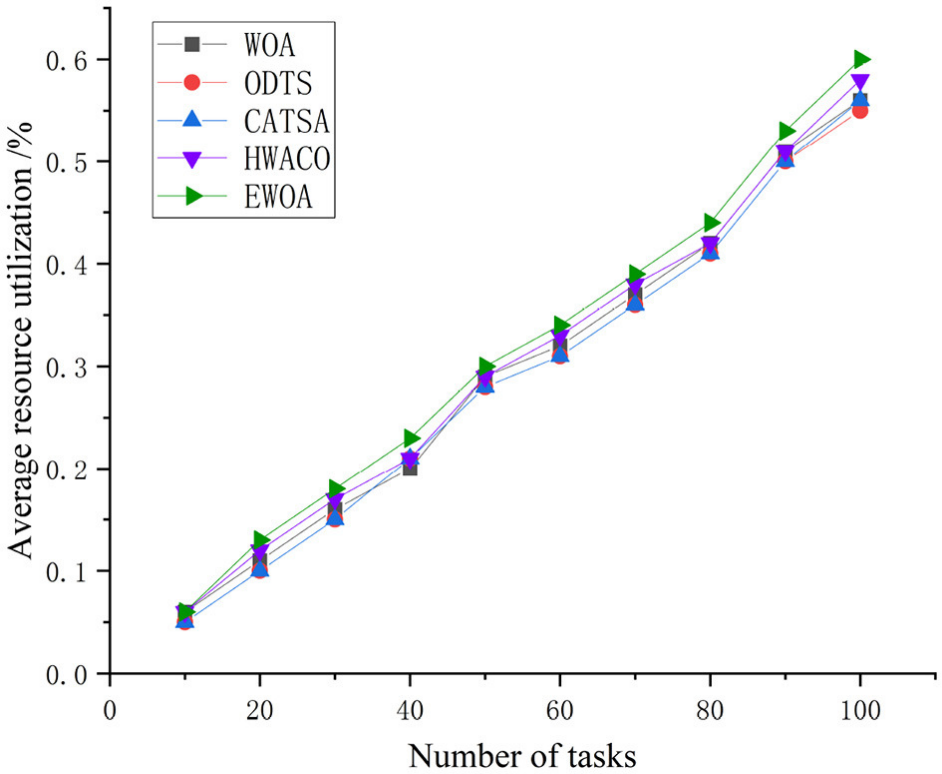
Comparison of resource utilization during the execution of small-scale tasks.

The EWOA algorithm server resource utilization outperforms the other three algo-rithms. Figure 7 illustrates that the resource utilization of all four algorithms increases as the task number increases. This is attributed to the EWOA algorithm's effectiveness in the execution of small-scale tasks where the limited number of tasks contributes to convenient and fast task execution. The incorporation of chaotic mapping and nonlinear convergence factors further accelerates the solution search, enhancing the server's resource utilization for task execution.

#### 7.1.2 Analysis of experimental results under large-scale tasks

**Figure 8:**
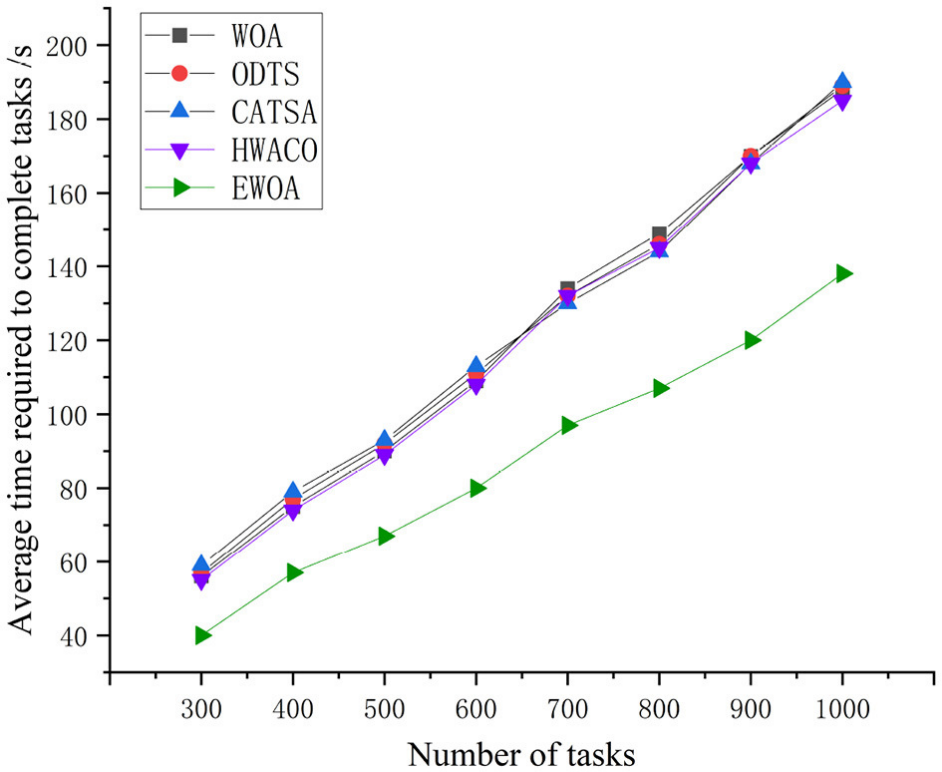
Comparison of task execution time during the performance of large-scale tasks.

Figure 8 clearly illustrates the execution times of the four algorithms, showing a smooth upward trend as the number of tasks increases. Among these, the EWOA algorithm stands out, reducing average execution time by 27.29, 27.74, 27.66, and 26.15% compared to WOA, ODTS, CATSA, and HWACO, respectively. This significant improvement can be attributed to the integration of the Tent chaos mapping in the EWOA algorithm. By incorporating this mapping, the whale algorithm is able to avoid local optima during later iterations, effectively overcoming the original whale algorithm's tendency to get trapped in suboptimal solutions. This, in turn, shortens the time required to find the optimal server, leading to the enhanced performance of the EWOA algorithm.

**Figure 9:**
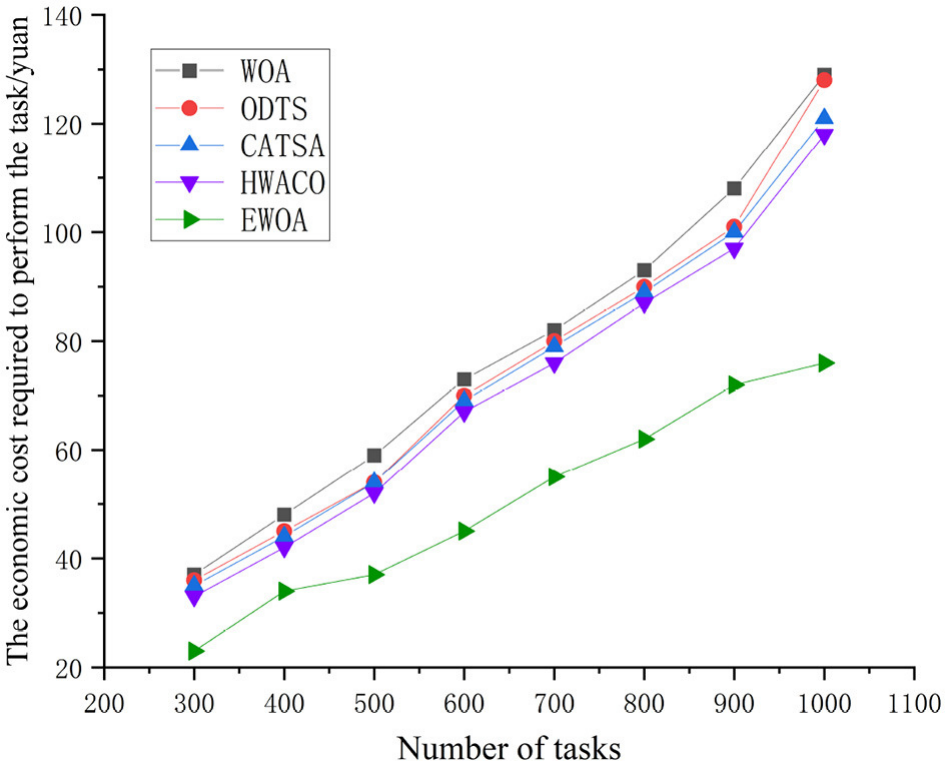
Comparison of task execution cost during the performance of large-scale tasks.

As depicted in Figure 9, the task execution cost rises as the task count reaches 1,000. Notably, the WOA, ODTS, CATSA, and HWACO algorithms exhibit higher rates of cost escalation compared to the EWOA algorithm. In contrast to the WOA, ODTS, CATSA, and HWACO algorithms, the EWOA algorithm achieves a reduction in economiccosts by 38.9, 38.4, 34.9, and 33.8%, respectively. This reduction is attributed to the EWOA algorithm's capability to address edge overloading issues by efficiently distributing tasks with the cloud during the execution of large-scale jobs when resources at the edge are limited. Additionally, the EWOA algorithm effectively addresses the issue of getting trapped in local optima that existed in the original algorithm. This improvement leads to lower task execution costs. It is evident that the EWOA algorithm offers several advantages over the other algorithms.

**Figure 10:**
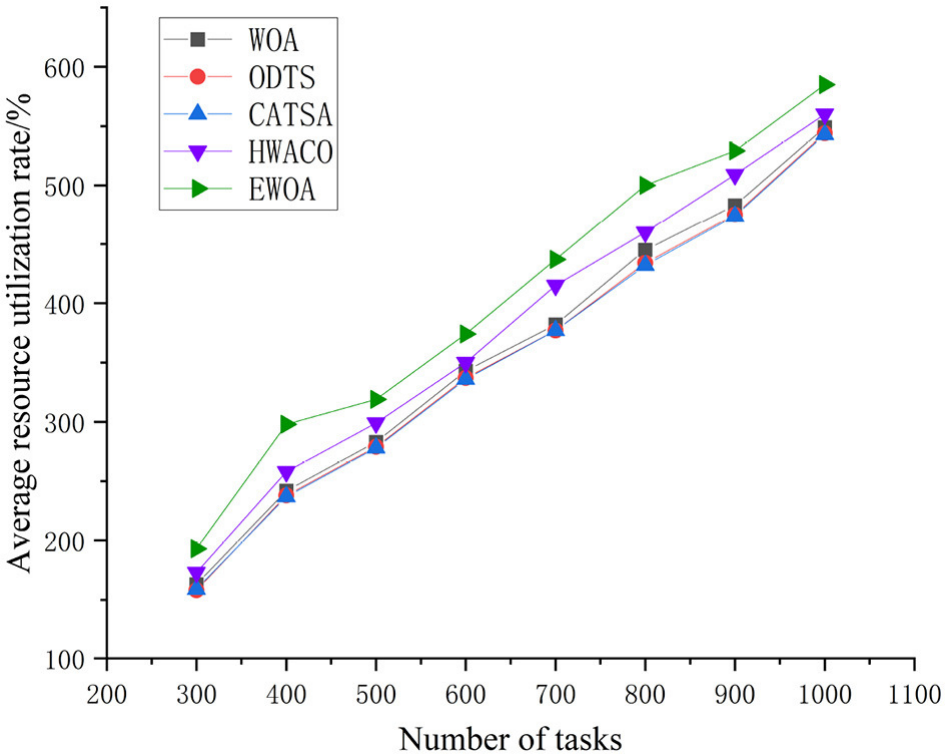
Comparison of resource utilization during the execution of large-scale tasks.

Figure 10 unequivocally illustrates that the EWOA algorithm surpasses the other three algorithms in resource utilization, especially evident during the execution of large-scale tasks. This is because of the successful integration of chaotic mapping and non-linear convergence factors within the algorithm. This synergy enhances the algorithm's capacity for global-scale search, effectively reconciles global and local search conflicts, and addresses the common convergence issues seen in traditional whale algorithms. These traditional algorithms often converge slowly and tend to get trapped in local optima. The EWOA algorithm minimizes server resource wastage and overutilization by systematically searching for the optimal location to process each task, resulting in a reduction in overall resource utilization.

After a thorough analysis of six sets of experimental data, our study demonstrates that the proposed EWOA algorithm presents significant advantages in both cost and task execution time. This superiority is particularly pronounced in the context of scheduling tasks within edge cloud computing environments. Additionally, the observed stability of the benefit function across iterations indicates that EWOA converges reliably toward optimal solutions. The consistent performance observed across multiple runs suggests that the algorithm effectively avoids premature convergence, allowing for robust optimization. This reliable convergence behavior further supports the success rate of EWOA in addressing the task scheduling problem in edge computing environments.

### 7.2 Comparison of algorithms

To further quantify the distinctions between the proposed EWOA algorithm and the baseline methods (WOA, ODTS, CATSA, and HWACO), effect sizes were computed using Cohen's d for pairwise comparisons across key performance metrics, including execution time, cost, and resource utilization. Effect size analysis via Cohen's d is critical for assessing the practical significance of observed differences, with benchmarks of 0.2, 0.5, and 0.8 indicating small, medium, and large effects, respectively. These evaluations are vital for demonstrating not only the statistical significance of the differences (as indicated by *P*-values) but also the magnitude of these differences in terms of real-world relevance.

As shown in Table 4, EWOA achieves notable improvements in cost efficiency, particularly in comparison to HWACO, with a Cohen's d of –0.74, demonstrating significant cost savings. While the effect sizes for execution time are more modest, ranging from –0.09 to –0.17, EWOA still offers measurable enhancements in task completion time across all comparisons. Additionally, the resource utilization results, with Cohen's d values reaching 0.26 against WOA, suggest that EWOA provides more efficient use of resources, contributing to a well-balanced overall performance.

**Table 4:** Cohen's d effect size results for EWOA compared to other algorithms.

**Algorithm comparison**	**Cohen's d (execution time)**	**Cohen's d (cost)**	**Cohen's d (resource utilization)**
EWOA vs. WOA	-0.15	-0.61	0.26
EWOA vs. ODTS	-0.17	-0.48	0.17
EWOA vs. CATSA	-0.17	-0.46	0.18
EWOA vs. HWACO	-0.09	-0.74	0.09

As shown in Table 5, EWOA demonstrates substantial improvements in cost efficiency, with Cohen's d values reaching –1.22 when compared to HWACO and –1.10 against WOA, indicating significant cost reductions in large-scale tasks. In terms of execution time, EWOA continues to show improvements, with Cohen's d values around –0.25 to –0.27, reflecting consistent but moderate enhancements in task completion time. For resource utilization, EWOA also provides beneficial performance, with Cohen's d values ranging from 0.21 to 0.38, highlighting its effectiveness in optimizing resource use across a larger number of tasks.

**Table 5:** Cohen's d effect size results for EWOA compared to other algorithms.

**Algorithm comparison**	**Cohen's d (execution time)**	**Cohen's d (cost)**	**Cohen's d (resource utilization)**
EWOA vs. WOA	–0.27	–1.10	0.32
EWOA vs. ODTS	–0.27	–0.98	0.37
EWOA vs. CATSA	–0.27	–0.91	0.38
EWOA vs. HWACO	–0.25	–1.22	0.21

The analysis of both task sizes demonstrates that EWOA is particularly effective in optimizing costs for large-scale tasks. It also delivers solid performance in resource utilization and execution time. Notably, the medium effect size observed in resource utilization suggests that EWOA achieves higher efficiency when managing larger task loads.

## 8 Conclusion

The increasing reliance on mobile devices and compute-intensive applications has introduced significant challenges in edge computing environments, particularly in terms of limited resources and the need for efficient task scheduling. In response, this study introduced an enhanced whale optimization algorithm (EWOA) specifically designed for task scheduling in edge computing. By utilizing chaotic mapping, the algorithm enhances search accuracy and mitigates premature convergence, while the incorporation of a nonlinear convergence factor ensures a balanced approach between global and local search, improving the overall optimization process. The experimental results validate the effectiveness of EWOA, demonstrating superior performance compared to ODTS, WOA, HWACO, and CATSA algorithms. EWOA achieved a 29.22% reduction in task scheduling costs, a 17.04% decrease in average task completion time, and a 9.5% improvement in resource utilization. Despite these advantages, the current implementation has some limitations. It does not fully consider potential network delays and the challenges posed by user mobility, which may lead to reduced performance in highly dynamic edge environments. Future research will focus on overcoming these limitations by developing more resilient and fault-tolerant scheduling techniques that can adapt to real-time changes and improve the quality of service in edge computing scenarios.

## Data Availability

The datasets presented in this study can be found in online repositories. The names of the repository/repositories and accession number(s) can be found at: https://snap.stanford.edu/data.
